# A Single Nucleotide Polymorphism near the *CYP17A1* Gene Is Associated with Left Ventricular Mass in Hypertensive Patients under Pharmacotherapy

**DOI:** 10.3390/ijms160817456

**Published:** 2015-07-30

**Authors:** Matthias Huber, Susanne Lezius, Rona Reibis, Andras Treszl, Dorota Kujawinska, Stefanie Jakob, Karl Wegscheider, Heinz Völler, Reinhold Kreutz

**Affiliations:** 1 Institute of Clinical Pharmacology and Toxicology, Charité–Universitätsmedizin Berlin, 10117 Berlin, Germany; E-Mails: dorotakuj@yahoo.co.uk (D.K.); stefanie.jakob@charite.de (S.J.); reinhold.kreutz@charite.de (R.K.); 2 Department of Medical Biometry and Epidemiology, University Medical Center Hamburg-Eppendorf, 20246 Hamburg, Germany; E-Mails: s.lezius@uke.de (S.L.); a.treszl@uke.uni-hamburg.de (A.T.); k.wegscheider@uke.de (K.W.); 3 Cardiological Outpatient Clinic Am Park Sanssouci, 14469 Potsdam, Germany; E-Mail: rona.reibis@hotmail.de; 4 Klinik am See, Rehabilitation Center for Cardiovascular Diseases, 15562 Rüdersdorf, Germany; E-Mail: voeller@uni-potsdam.de; 5 Center of Rehabilitation Research, University of Potsdam, 14469 Potsdam, Germany

**Keywords:** clinical study, genetics, heart, hypertension, cytochrome P450 17A1 (Cyp17A1)

## Abstract

Cytochrome P450 17A1 (CYP17A1) catalyses the formation and metabolism of steroid hormones. They are involved in blood pressure (BP) regulation and in the pathogenesis of left ventricular hypertrophy. Therefore, altered function of CYP17A1 due to genetic variants may influence BP and left ventricular mass. Notably, genome wide association studies supported the role of this enzyme in BP control. Against this background, we investigated associations between single nucleotide polymorphisms (SNPs) in or nearby the CYP17A1 gene with BP and left ventricular mass in patients with arterial hypertension and associated cardiovascular organ damage treated according to guidelines. Patients (*n* = 1007, mean age 58.0 ± 9.8 years, 83% men) with arterial hypertension and cardiac left ventricular ejection fraction (LVEF) ≥40% were enrolled in the study. Cardiac parameters of left ventricular mass, geometry and function were determined by echocardiography. The cohort comprised patients with coronary heart disease (*n* = 823; 81.7%) and myocardial infarction (*n* = 545; 54.1%) with a mean LVEF of 59.9% ± 9.3%. The mean left ventricular mass index (LVMI) was 52.1 ± 21.2 g/m^2.7^ and 485 (48.2%) patients had left ventricular hypertrophy. There was no significant association of any investigated SNP (rs619824, rs743572, rs1004467, rs11191548, rs17115100) with mean 24 h systolic or diastolic BP. However, carriers of the rs11191548 C allele demonstrated a 7% increase in LVMI (95% CI: 1%–12%, *p* = 0.017) compared to non-carriers. The CYP17A1 polymorphism rs11191548 demonstrated a significant association with LVMI in patients with arterial hypertension and preserved LVEF. Thus, CYP17A1 may contribute to cardiac hypertrophy in this clinical condition.

## 1. Introduction

Cytochrome P450 17A1 (CYP17A1) is a key enzyme in the synthesis and metabolism of steroid hormones. As a unique protein of the cytochrome P450 family it catalyzes two distinct types of substrate oxidation [1,2]. This includes steroid 17alpha-hydroxylation activity, which is essential for the biosynthesis of corticoids and the 17, 20 lyase reaction which generates precursors of sex steroids [3,4,5,6,7]. CYP17A1 is encoded by a single gene on chromosome 10q24.32 and consists of eight exons and seven introns [6,7].

In the clinical context, CYP17A1 has primarily emerged as relevant for androgen dependent oncological diseases [8,9,10]. In particular, prostate cancer is influenced by the activity and genetics of this enzyme and a specific CYP17A1 inhibitor was recently approved by the Food and Drug Administration for this indication [2,11,12]. Furthermore, genetic association studies suggest that CYP17A1 plays a role in different pathological conditions such as in Parkinson’s disease [13], Alzheimer’s disease [14] or obesity [15].

The evidence supporting a role of CYP17A1 in the cardiovascular field is still scarce. Notably, the first case report of 17alpha hydroxylase deficiency, published in the year 1966, already pointed to hypertension as a phenotypic characteristic [16]. Concordant with this previous observation, hormonal substances such as corticoids and sex steroids are increasingly considered as important factors in the development of hypertension and the related target organ damage [17,18,19,20]. This substantiates a rationale for CYP17A1 as an important enzyme in the pathogenesis of both conditions. Notably, results of genome wide association studies (GWAS) support this notion indicating significant relations of single nucleotide polymorphisms (SNPs) in or nearby CYP17A1 gene to blood pressure (BP) parameters [21,22,23,24,25,26,27]. These analyses were based on large populations with or without cardiovascular diseases and therapies [28].

The present study focused on 1007 patients with arterial hypertension and associated cardiovascular organ damage that were treated according to European guidelines [29]. We tested genetic associations of SNPs in or nearby CYP17A1 with 24 h BP levels and left ventricular mass in this population.

## 2. Results

### 2.1. Description of the Study Cohort

The characteristics of the study cohort are summarized in Table 1. We studied 1007 patients, 834 (82.8%) men and 173 (17.2%) women with a mean age of 58.0 ± 9.8 years. The mean 24 h systolic and diastolic BP values were 125.0 ± 14.7 and 73.8 ± 9.5 mmHg, respectively. Overall, 823 (81.7%) patients had coronary heart disease and 545 (54.1%) subjects had a history of myocardial infarction at least one month before enrolment in the study. The most commonly used antihypertensive drugs were beta-blockers (*n* = 883; 87.7%) and angiotensin-converting enzyme inhibitors (*n* = 738; 73.3%).

**Table 1:** Characteristics of study cohort (*N* = 1007).

Parameter	Value
Age (years)	58.0 ± 9.8
Men	834 (82.8%)
Women	173 (17.2%)
BMI (kg/m^2^)	28.9 ± 4.7
Current smoker	257 (25.5%)
eGFR * (mL × min^−1^ × 1.73 m^−2^)	78.6 ± 21.0
eGFR < 60 (mL × min^−1^ × 1.73 m^−2^)	135 (13.4%)
Coronary heart disease	823 (81.7%)
Myocardial infarction	545 (54.1%)
Diabetes mellitus	270 (26.8%)
**Mean 24 h BP (mmHg)**	
systolic	125.0 ± 14.7
diastolic	73.8 ± 9.5
**Antihypertensive drugs**	
ACE inhibitors	738 (73.3%)
AT1-antagonists	155 (15.4%)
beta-blockers	883 (87.7%)
calcium antagonists	142 (14.1%)
diuretics	436 (43.3%)
other drugs	55 (5.5%)

Data are given as mean ± standard deviation or as numbers and percentages in parentheses per total of 1007 subjects; * estimated glomerular filtration rate (eGFR) was calculated according to Levey *et al.* [30]: eGFR (mL/min per 1.73 m^2^) = 186 × (serum creatinine in mg/dL)^−1.154^ × (age in years)^−0.203^ × (0.742 if female) × (1.210 if African-American); ACE, angiotensin converting enzyme; AT1, angiotensin type 1 receptor.

### 2.2. Echocardiographic Parameters of Study Cohort

Echocardiographic parameters of the study cohort are demonstrated in Table 2. The mean left ventricular mass index (LVMI) was 52.1 ± 21.2 g/m^2.7^. Left ventricular hypertrophy defined as LVMI ≥ 50 g/m^2.7^ in men and ≥47 g/m^2.7^ in women was observed in 485 (48.2%) patients according to de Simone *et al.* [31]. The mean left ventricular ejection fraction (LVEF) was 59.9% ± 9.3% indicating that overall left ventricular systolic function was well preserved. Left atrium was slightly dilated (41.1 ± 5.4 mm) and internal left ventricular diastolic dimensions were in the normal range (51.1 ± 7.0 mm).

**Table 2:** Echocardiographic parameters of study cohort (*N* = 1007).

Parameter	Value
LVMI (g/m^2.7^) overall *	52.1 ± 21.2
men	52.2 ± 21.7
women	51.6 ± 18.4
Left ventricular hypertrophy overall ^†^	485 (48.2%)
men	390 (46.8%)
women	95 (54.9%)
LVEF (%)	59.9 ± 9.3
LA (mm)	41.1 ± 5.4
LVED (mm)	51.1 ± 7.0
LVES (mm)	34.2 ± 7.1
E/A	1.13 ± 0.42
IVST (mm)	11.3 ± 2.7
PWT (mm)	11.0 ± 2.8
RWT	0.45 ± 0.16

Data are given as mean ± standard deviation or as numbers and percentages in parentheses per total of 1007 subjects; * LVMI, left ventricular mass index according to Baessler *et al.* [32]; ^†^ LVH, left ventricular hypertrophy according to de Simone *et al.* [31] definitions LVMI ≥ 50 g/m^2.7^ in men and ≥47 g/m^2.7^ in women; LA, left atrial diameter; LVED, left ventricular end-diastolic diameter; LVES, left ventricular end-systolic diameter; LVEF, left ventricular ejection fraction; E/A, ratio of early filling velocity (E) and peak late filling velocity (A); IVST, interventricular septum thickness; PWT, posterior wall thickness; RWT, relative wall thickness.

### 2.3. Genetic Analysis

The polymorphisms rs619824, rs743572, rs1004467, rs11191548, and rs17115100 were analysed for their relations to mean systolic and diastolic 24 h BP and LVMI. Allele and genotype frequencies are indicated in Supplemental Table S1. They were in agreement with data from the National Center for Biotechnology Information SNP databases. All genotype frequencies were consistent with the Hardy-Weinberg equilibrium.

#### 2.3.1. Analysis of Polymorphisms in Relation to 24 h BP Parameters

Multivariate adjusted analyses resulted in no significant associations of any investigated SNP with mean 24 h systolic or diastolic BP. Further separate analysis for mean day-time or night-time blood pressure phenotypes also demonstrated no significant associations, respectively (not shown).

#### 2.3.2. Analysis of Polymorphisms in Relation to LVMI

Results of multivariate adjusted analyses are presented in Table 3. For rs11191548 carriers of the C allele indicated compared to non-carriers a 7% increase in LVMI (95% CI: 1%–12%, *p* = 0.017). In analogue comparison the T allele of rs17115100 exhibited a trend to increased LVMI (*p* = 0.059). Correlation analyses of the SNP alleles with the use of betablockers or angiotensin-converting enzyme inhibitors in patients with LVH led to no significant results.

**Table 3:** Relation of single nucleotide polymorphisms (SNPs) with left ventricular mass index (LVMI) in stepwise multivariate adjusted analysis according to combined genotypes.

SNP Region *	SNP ID	Comparison	LVMI Ratio [95% CI]	*p* **
3ʹUTR	rs619824	CC + CA *vs.* AA	0.96 [0.91–1.01]	0.119
3ʹUTR	rs619824	CC *vs.* CA + AA	1.01 [0.96–1.06]	0.794
5ʹUTR(-34T/C)	rs743572	AA + AG *vs.* GG	0.96 [0.91–1.02]	0.186
5ʹUTR(-34T/C)	rs743572	AA *vs.* AG + GG	1.01 [0.97–1.06]	0.558
Intron 3	rs1004467	AA + AG *vs.* GG	0.95 [0.78–1.14]	0.569
Intron 3	rs1004467	AA *vs.* AG + GG	0.95 [0.91–1.01]	0.080
3ʹUTR	rs11191548	TT + TC *vs.* CC	1.02 [0.83–1.25]	0.872
3ʹUTR	rs11191548	TT *vs.* TC + CC	0.93 [0.88–0.99]	0.017
Intron 6	rs17115100	GG + GT *vs.* TT	0.94 [0.78–1.13]	0.496
Intron 6	rs17115100	GG *vs.* GT + TT	0.95 [0.90–1.00]	0.059

LVMI difference, e.g., for rs619824, carriers of C allele had a 0.96-fold LVMI compared to non-carriers; 95% CI, 95% confidence interval; * SNP region related to CYP17A1 gene; UTR, untranslated region; ** *p*-values of the corresponding ANCOVA model; models were adjusted for gender, age, BMI, height, eGFR, coronary heart disease, pharmacotherapy with oral anticoagulants, and laboratory findings LDL, triglycerides.

## 3. Discussion

The present study aimed to investigate genetic associations of variants in or nearby the CYP17A1 gene with 24 h BP and left ventricular mass in treated high risk patients with arterial hypertension and associated cardiovascular organ damage. Thus, about four out of five patients had coronary heart disease and about a half had myocardial infarction at least one month before enrolment in the study. However, in agreement with the inclusion criteria in the study, left ventricular function was well preserved with a minimum LVEF of 40% in each patient and an overall mean LVEF of about 60% observed in the study. About half of all patients demonstrated left ventricular hypertrophy (LVH) defined as LVMI ≥ 50 g/m^2.7^ in men or ≥47 g/m^2.7^ in women [31].

### 3.1. Relation of Polymorphisms to 24 h BP Parameters

In the present study, there was no significant association of any investigated SNP with mean 24 h systolic or diastolic BP. For two of the five investigated SNPs (rs11191548, rs1004467), significant relations to BP have previously been described. Thus, two genome wide association studies (GWAS) with cohorts each consisting of more than 29 thousand subjects of European ancestry and which were heterogeneous for the presence of hypertension and antihypertensive treatment revealed significant associations with increased systolic BP for the A allele of rs1004467 [24] and for the T allele of rs11191548 [25]. Studies with cohorts of East and South Asian origin replicated the latter finding and furthermore indicated an association of this allele with increased diastolic BP [21,22].

In parallel to the GWAS results, a Chinese study including 3210 unrelated individuals from Beijing and Shanghai described the A allele of rs1004467 and the T allele of rs11191548 as significantly related to increased risk of hypertension. In two case-control studies with Chinese children, analogous associations of both alleles with increased systolic BP were only found in girls [33] or in those subjects who were characterized as physical inactive by validated questionnaires [34].

Nevertheless, the relation of the rs1004467 A allele with increased systolic BP could not be replicated in a cohort of 3077 Chinese children [35]. In contrast to the GWAS results, a study in Chinese adults comparing 3292 hypertensive or pre-hypertensive subjects to 1168 normotensive controls revealed significant associations of the T allele of rs11191548 with decreased systolic and diastolic BP values [36]. In addition, a case control study including 1102 individuals with essential hypertension and 1109 normotensive controls of the same ethnic group described the C allele of rs11191548 as risk allele for increased systolic BP in the female, male and overall normotensive control groups. Remarkably, in parallel to the results of the present study, no significant association with BP was found in subjects with arterial hypertension [37].

Therefore, results of different studies about the associations of the SNPs in CYP17A1 gene with BP are so far heterogeneous and seem to depend on the context of the study.

### 3.2. Relation of Polymorphisms to LVMI

Notably, in the present study, the C allele of rs11191548 was significantly related to a 7% increase in LVMI compared to non-carriers. Furthermore, rs17115100 indicated a trend of the T allele towards elevated LVMI. These findings are novel and emerged in a typical clinical setting because patients with arterial hypertension were under pharmacotherapy according to guidelines.

Left ventricular mass may be considered as a parameter that integrates BP levels for the long-term and which is more resistant against transient influences than BP values. Moreover, increased LVMI above the cut-off values (50 g/m^2.7^ for men, 47 g/m^2.7^ for women [31]) constitutes LVH which is an independent and powerful risk factor for cardiovascular morbidity and mortality particular in patients with arterial hypertension [38,39]. Notably, the present finding resulted from a cohort in which about the half of patients indicated LVH that may implicate a role of CYP17A1 in the pathogenesis of cardiac target organ damage.

At the molecular level, CYP17A1 catalyses the metabolic pathway from pregnenolone to 17alpha hydroxy-pregnenolone which is an intermediate substance in the synthesis of cortisol and sex steroids. Competitive to this synthetic pathway pregnenolone can be metabolized to aldosterone [40]. The molecular mechanism which underlies the genetic association of the rs11191548 C allele with increased LVMI is still unknown and will be an interesting topic for experimental studies. Nevertheless, one may speculate that the genetic variant rs11191548 may be involved in the regulation of the transcription of CYP17A1 with subsequent influences on the disposition of sex steroids, cortisol and aldosterone. In line with this concept are clinical reports which describe remarkable changes of levels of these hormones in patients with disorders of CYP17A1 [41,42,43]. Aldosterone is a potent molecule in the regulation of cell growth and survival [44]. Thus, aldosterone is able to induce hypertrophy of cardiomyocytes *in vitro* including the expression of hypertrophic markers such as A- or B-type natriuretic peptides (ANP, BNP) or cardiotrophin-1 [45,46]. Therefore, aldosterone is considered as one of the important humoral factors in the pathogenesis of LVH [17,47].

Clinical studies consistently indicated positive correlations between plasma aldosterone levels and left ventricular mass in hypertensive patients [48,49,50,51,52]. Moreover, aldosterone receptor antagonists reduced LVMI in hypertensive patients with left ventricular hypertrophy [53]. Cortisol has been described as major determinant of LVH in Cushing’s syndrome [19]. In untreated hypertensive patients LVMI correlated significantly with 24 h urinary free cortisol and cortisone [18]. Finally, sex steroids, in particular androgens contribute to higher left ventricular mass in men compared to women and are involved in gender specific progress of cardiac hypertrophy [20]. The steroid hormone dehydroepiandrosterone is metabolized via 16alpha hydroxylation by CYP17A1 [40,54] and prevented hypertrophy of cardiomyocytes in animal studies [45].

Overall, these experimental and clinical studies point to CYP17A1 as a key enzyme in the generation and metabolism of humoral factors which are involved in the pathogenesis of LVH. Accordingly, CYP17A1 has been discussed as drug target for treatment of hypertensive target organ damage [40].

### 3.3. Possible Clinical Implications

The present study describes the SNP rs11191548 as significantly associated with left ventricular mass in patients with hypertension under pharmacotherapy. Therefore, this SNP may be a relevant marker for the risk to develop LVH in an individual patient. Consequently, screening of patients may offer the possibility for a more personalized medicine in the future including the early onset of preventive strategies. Nevertheless, further studies are necessary to implement this concept.

### 3.4. Limitations of the Study

This study has important limitations. Left ventricular mass was calculated according to the American Society of Echocardiography-recommended formula for estimation of left ventricular mass from left ventricular linear dimensions [55]. The formula is appropriate for evaluating patients without major distortions of left ventricular geometry. To avoid incurring errors due to substantially distorted ventricles patients with marked segmental left ventricular dysfunction were not enrolled in the cohort. Marked left ventricular dysfunction was defined as akinesia or dyskinesia of two or more segments of the 16 segment model of the left ventricle. This included left ventricular aneurysma, asymmetric dilatation and mass distribution such as post-infarctional regional wall thinning. Furthermore, we a priori excluded patients with an LVEF < 40%. Only investigators with a long lasting experience in echocardiography were accredited to data acquisition and underwent joint training prior to the study where standards were defined and practically rehearsed. Therefore, in parallel to other large-scale genetic investigations [56] it can be expected that the measurement of LVMI by echocardiography was adequate to allow a reliable genetic analysis in the current context. Nevertheless, the present finding must be interpreted against the background of the used technique in phenotyping and should be confirmed by further clinical investigations.

The presented results raise the hypothesis that rs11191548 may influence the activity of CYP17A1. Nevertheless the underlying molecular mechanisms are so far unclear and should be clarified by experimental studies.

## 4. Patients and Methods

### 4.1. Study Population and Clinical Evaluation

In this study 1007 Caucasian subjects (173 women, 17.2%) who participated in a cardiological inpatient rehabilitation program were analysed. All patients had a diagnosis of arterial hypertension according to guidelines criteria defined as average BP of at least 140 mmHg systolic or at least 90 mmHg diastolic [29]. All patients were treated according to European guidelines and had a history of at least 1 month of documented cardiovascular index event including myocardial infarction at least one month before enrolment in the study. Each patient was interviewed by a standard procedure including demographic data, medical history and medication. All subjects were examined by a physician and ambulatory 24 h BP measurements were taken with automatic portable devices (custo med GmbH Ottobrunn, Germany) every 15 min during the day (defined from 6 to 22 h) and every 30 min during the night. Echocardiography was performed according to established standards [31,55,57,58] and is described in the supplemental digital content. Blood samples were collected after a 12 h fasting period and analysed with standard procedures of clinical chemistry. Estimated glomerular filtration rate (eGFR) was calculated according to Levey *et al.* [30]: eGFR (mL/min per 1.73 m^2^) = 186 × (serum creatinine in mg/dL)^−1.154^ × (age in years)^−0.203^ × (0.742 if female) × (1.210 if African-American). This study complies with the Declaration of Helsinki, written informed consent was obtained from all subjects, and the local Ethics Committee of the Campus Benjamin Franklin (Charité—Universitätsmedizin, Berlin, Germany) approved the study protocol.

### 4.2. Determination of Genotypes

Genotyping for CYP17A1 polymorphisms was performed by PCR with the fluorescence based TaqMan^®^ system (Applied Biosystems, Darmstadt, Germany) with the pre-designed tested assays from the manufacturer. More details are described in the supplemental digital content. Our overall combined genetic analyses included the independent analysis of recessive (YY *vs.* YX plus XX) and dominant (YX plus YY *vs.* XX) models.

### 4.3. Statistical Analysis

Parameters, indicated in Table 1 and Table 2, were analysed by methods of descriptive statistics and data are presented as numbers and percentages per total of 1007 subjects or as arithmetic means ± standard deviations. The distribution of the data was checked and if data were not normally distributed, data were log transformed to reach normal distribution. Following statistical analyses, all log transformed values were back-transformed. Analyses of covariance models were calculated and the results are reported as mean and 95% confidence intervals of the mean (CI). Analyses of covariance models were applied to investigate the simultaneous influence of potential confounding factors on BP and the echocardiographic parameter. In the stepwise multivariate analysis regressors were entered blockwise into the model. First, the demographic variables (age, gender and (if significant) their interaction) were entered then, in three blocks variables were added (block 1: cardiac status, block 2: pharmacological treatment, and block 3: laboratory findings, respectively); after analysis in each block a final backward selection was performed. Variables selected in the last step are reported. A confidence-limit-based approach was applied to the assessment of Hardy-Weinberg equilibrium. Two-tailed values of *p* less than 0.05 were considered statistically significant. Correlation analyses were performed using chi-squared tests or Fisher’s exact test, as appropriate. *p*-values below 0.05 were considered significant.All statistical analyses were carried out using SAS 9.2 (SAS-Institute, Cary, NC, USA) or SPSS 20 (SPSS Incorporation, Chicago, IL, USA).

## 5. Conclusions

As conclusion, the CYP17A1 polymorphism rs11191548 has been identified as associated with LVMI in high risk patients with arterial hypertension and associated organ damage. The result supports a role of CYP17A1 in the modulation of LVMI and thus cardiac hypertrophy in this clinical condition.

## Supplementary Materials

Supplementary materials can be found at http://www.mdpi.com/1422-0067/16/08/17456/s1.
