# Supplementary material for: A Single Nucleotide Polymorphism near the CYP17A1 Gene Is Associated with Left Ventricular Mass in Hypertensive Patients under Pharmacotherapy
Source: Int J Mol Sci. 2015 Jul 30;16(8):17456–68. doi: 10.3390/ijms160817456 (PMC4581202; doi:10.3390/ijms160817456)
Supplement: Supplementary file 1 [file ijms-16-17456-s001.pdf]

Supplementary Information

Supplemental Methods

Echocardiography

Two-dimensional guided M-mode echocardiography was performed for the determination of left ventricular hypertrophy (LVH) and geometry parameters. Left ventricular ejection fraction was calculated by biplane Simpson's rule method, as recommended in current guidelines [55]. Left ventricular mass (LVM) was calculated according to the American Society of Echocardiography-recommended formula for estimation of LVM from left ventricular linear dimensions [55], using M-mode measurements. For ejection fraction as well as mass calculations, values from three beats in sinus rhythm and five beats in atrial fibrillation were averaged.

To fulfil the assumption of symmetric left ventricular shape underlying LVM determination by M-mode echocardiography, patients with marked segmental left ventricular dysfunction were not enrolled in our cohort. Marked left ventricular dysfunction was defined as akinesia or dyskinesia of two or more segments of the 16 segment model of the left ventricle. This includes left ventricular aneurysma, asymmetric dilatation and mass distribution (postinfarctional regional wall thinning). An ejection fraction less than 40% was an exclusion criterion. To compare the LVM among patients, we used the left ventricular mass index (LVMI) normalized to body height$^{2.7}$ in g/m$^{2.7}$ [58]. LVH was defined as an LVMI $\geq$ 47 g/m$^{2.7}$ in women and LVMI $\geq$ 50 g/m$^{2.7}$ in men [31]. Relative wall thickness (RWT) was determined as an index of the geometric pattern of hypertrophy [57]. In addition, we determined the left atrium diameter (LA), left ventricular end-systolic diameter (LVES) and the E/A ratio.

All echocardiographic measurements were performed by one experienced operator at each center. All operators involved in the study underwent joint training at a workshop prior to the study where standards were defined and practically rehearsed. The responsible echo operators at each center made a decision which data were of sufficient quality for further analysis, based on overall quality and exclusion criteria. Echocardiographic images were digitally stored and analysed at each center off-line and blinded to other clinical data and the genotype of the patients.

Determination of genotypes

Genotyping of selected polymorphism was performed by PCR with fluorescence detection. The ABI PRISM 7000 SDS instrument in conjunction with the ABI TaqMan Universal Master Mix® (Applied Biosystems, Foster City, CA, USA) was used to perform the assays. Appropriate primers and the fluorogenic probes were obtained as pre-designed tested assays from the manufacturer (Applied Biosystems, Darmstadt, Germany). The TaqMan® assays were performed in 96 well plates (Perkin-Elmer, Applied Biosystems, Foster City, CA, USA). Each well contained a final PCR-reaction volume of 20 µL with 50 ng of genomic DNA, 400 nM primers, 100 nM probes and 10 µL of ABI TaqMan Universal PCR Master Mix® (Applied Biosystems, Foster City, CA, USA). Amplification was done under the following conditions: 50 °C, 2 min; 95 °C, 10 min; followed by 40 cycles of 94 °C, 15 s and 58 °C, 1 min. Data were analyzed using the ABI Prism 7000 SDS 1.0 Software (Perkin-Elmer, Applied Biosystems, Foster City, CA, USA). Genotyping was confirmed in 100 randomly selected probes by

direct DNA sequencing (BigDye® Terminator v3.1 Cycle Sequencing Kit, ABI PRISM 3100 Genetic Analyzer, Applied Biosystems, Darmstadt, Germany).

Table S1. Genotypes and minor allele frequencies of the study cohort.

SNP ID	N	Major Allele	Minor Allele	MAF	Minor Allele Homozygous	Heterozygous	Major Allele Homozygous
rs619824	1007	C	A	0.468	214 (21.3)	515 (51.1)	278 (27.6)
rs743572	1007	A	G	0.416	168 (16.7)	502 (49.9)	337 (33.5)
rs1004467	1007	A	G	0.107	13 (1.3)	190 (18.9)	804 (79.8)
rs11191548	1007	T	C	0.090	11 (1.1)	159 (15.8)	837 (83.1)
rs17115100	1007	G	T	0.107	14 (1.4)	187 (18.6)	806 (80.0)

N, number of genotypes; MAF, minor allele frequency; numbers in parenthesis indicate the relative portion of the respective genotype.

Appendix

Participating centers

The study was conducted in cooperation with the German Society for Prevention and Rehabilitation of Cardiovascular Diseases (DGPR e.V.). The following centers participated: Klinik am See, Rüdersdorf, Rona Reibis; Klinik Königsfeld, Königsfeld, Marthin Karoff; MediClin Reha-Zentrum Spreewald, Burg, Wolfram Kamke; Frankenklinik, Bad Neustadt an der Saale, Klaus Schröder; Montanus-Klinik, Bad Schwalbach, Thomas Gräf; Knappschafts-Klinik, Bad Driburg, Birgit Aue; Klinikzentrum Mühlengrund, Bad Wildungen, Sieglinde Spörl-Dönch; Fachklinik Sonnenhof, Waldachtal, Thomas Witt; Gollwitz-Meier-Klinik, Bad Oeynhausen, Karin Rosenblatt; Reha-Klinik Wehrawald der BfA, Todtmoos, Joachim Müller; Schüchtermann-Klinik, Bad Rothenfelde, Christian Baumbach; AmKaRe, Köln, Detlef Gysan; all in Germany.
